# Supplementary material for: Separation of sticker-spacer energetics governs the coalescence of metastable condensates
Source: Biophys J. 2024 Dec 15;124(2):428–39. doi: 10.1016/j.bpj.2024.12.017 (PMC11788481; doi:10.1016/j.bpj.2024.12.017)
Supplement: Document S1. Figures S1–S15 [file mmc1.pdf]

Biophysical Journal, Volume *124*

Supplemental information

Separation of sticker-spacer energetics governs the coalescence of metastable condensates

Aniruddha Chattaraj and Eugene I. Shakhnovich

Supplementary Figures

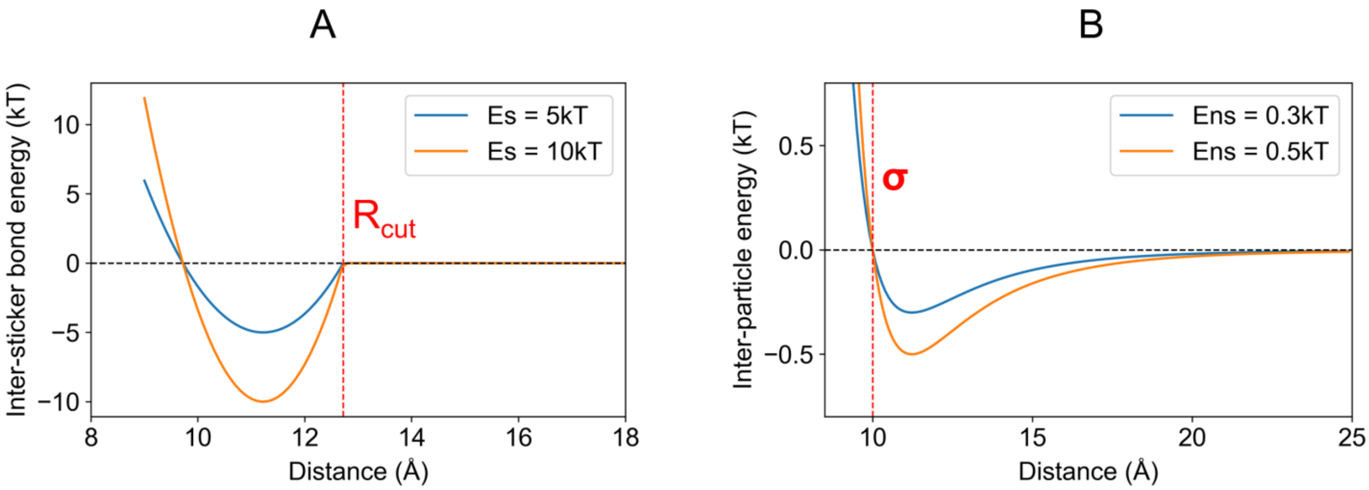

Figure S1: Illustration of specific and non-specific energy potentials. (A) The inter-sticker bonds are modelled with a shifted harmonic potential which becomes zero at a distance greater than R_{cut}. At the resting bond distance, the gain in energy is Es ($E_{specific}$). In other words, the depth of energy potential is Es at the resting distance. Two energy potentials are depicted for two different Es. (B) Pairwise non-specific interactions are modelled with Lennard-Jones potential which enforces excluded volume by the parameter, σ. The energy minima lies at the resting distance, that is 1.122 * σ. Depth of the energy well is Ens ($E_{non\text{-}specific}$). Two potentials are depicted at two different Ens.

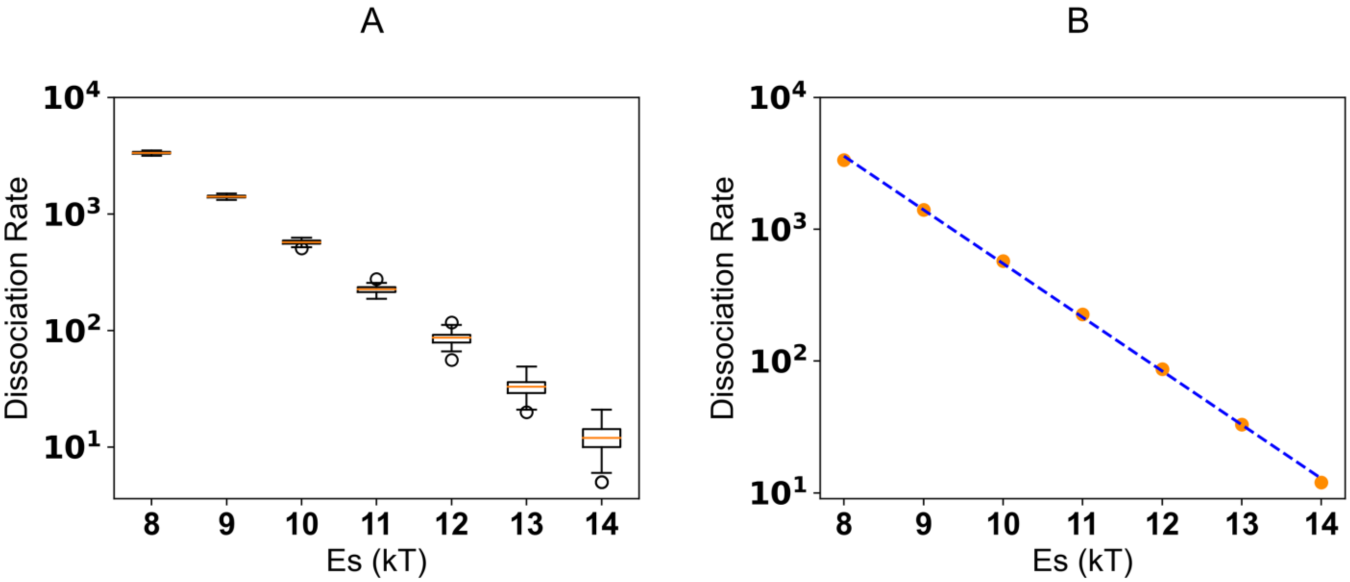

Figure S2: Inter-sticker dynamics follows an Arrhenius-like rate. For sticker-sticker interactions, rate of dissociation, $r \propto e^{-\frac{E_S}{kT}} \Rightarrow \log\,(r) \propto -\frac{Es}{kT}$. (A) The number of bond dissociation events between the red and cyan stickers (Figure 1A), at Ens = 0.3kT, as a function of specific interaction energy (Es). We note the log scale on the vertical axis. For each condition, we sampled 50 timeframes when the system is equilibrated. To display the distribution, we used standard boxplot representation or "five-number summary" consisting of the minimum, the maximum, the sample median, and the first and third quartiles. (B) The logarithm of dissociation events is fitted as a linear (blue dashed line) function of Es which yields a negative slope, consistent with an Arrhenius-like rate expression.

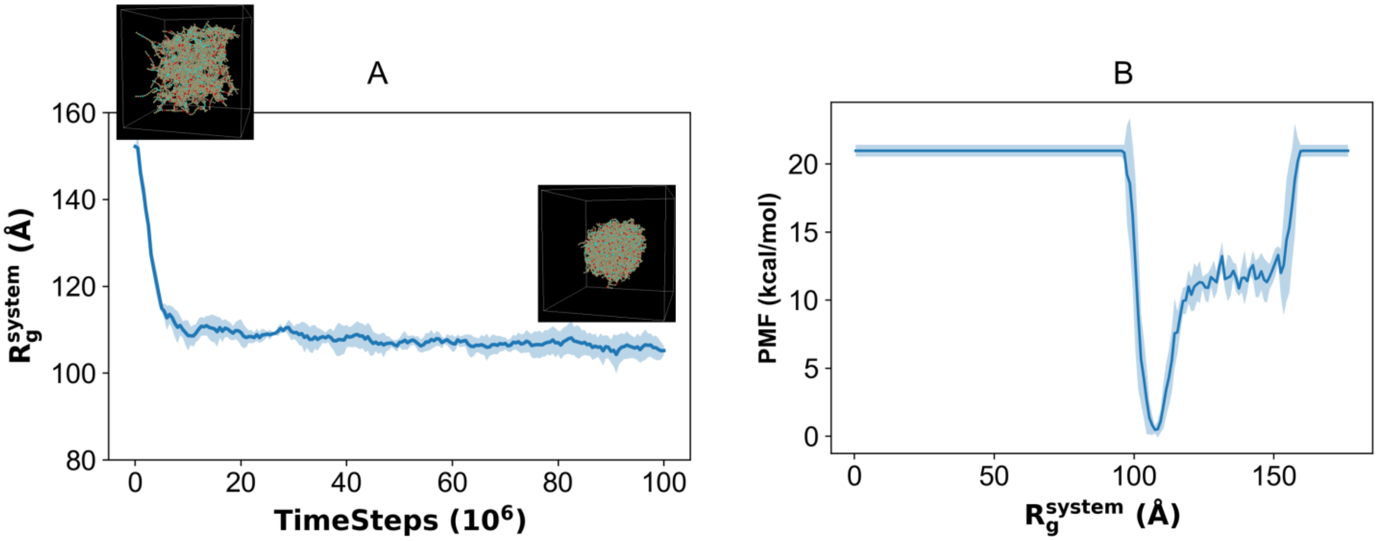

Figure S3: Coalescence dynamics of 200 chains into one large cluster. Energy parameters, Es = 10kT, Ens = 0.5kT. (A) Timecourse of the metadynamics order parameter R_g^{system}. Insets show first and last timeframes depicting dispersed and clustered states. (B) Free energy profile where the minimum corresponds to the fully clustered state. Each line is an average of 5 stochastic trials. The solid line is the mean and fluctuation envelop represents the standard deviation.

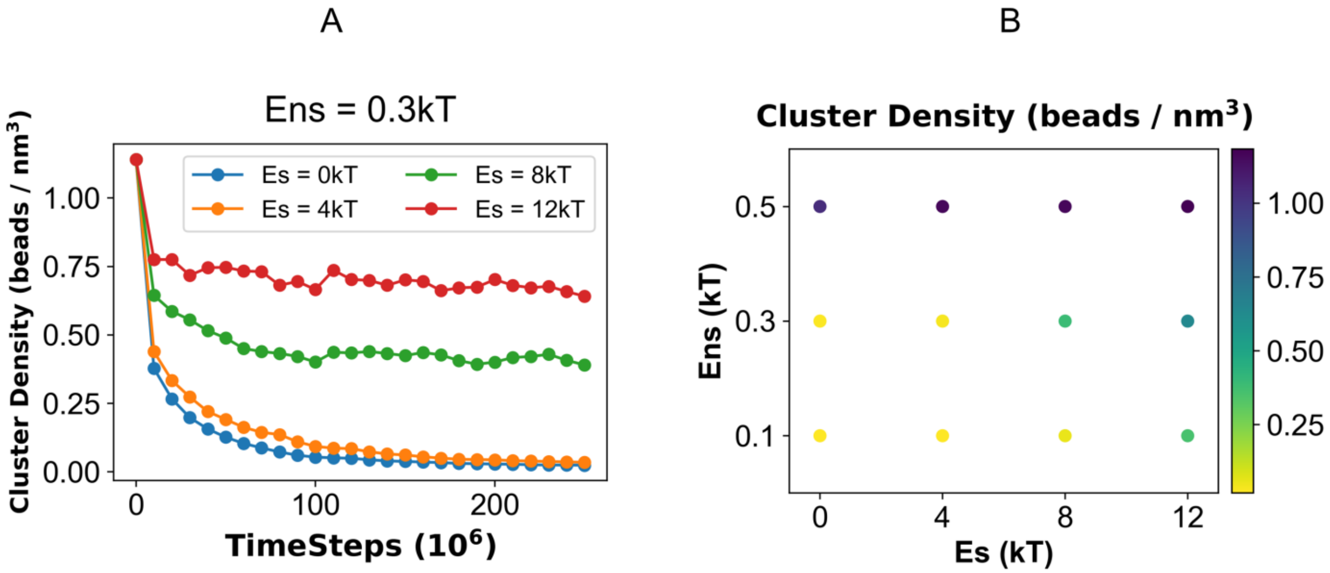

Figure S4: Quantification of phase transition boundary from the relaxation dynamics. (A) Timecourse of the cluster density, for a fixed Ens = 0.3kT, as a function of specific interaction strength, Es. (B) Phase diagram of the cluster density which is computed at the last timepoint of relaxation trajectory. $Density = \frac{N_{beads}}{\frac{4}{3}*\pi*R_g^3}$ where R_g is radius of gyration of the cluster and N_{beads} is the total number of beads (stickers + spacers) present in the cluster.

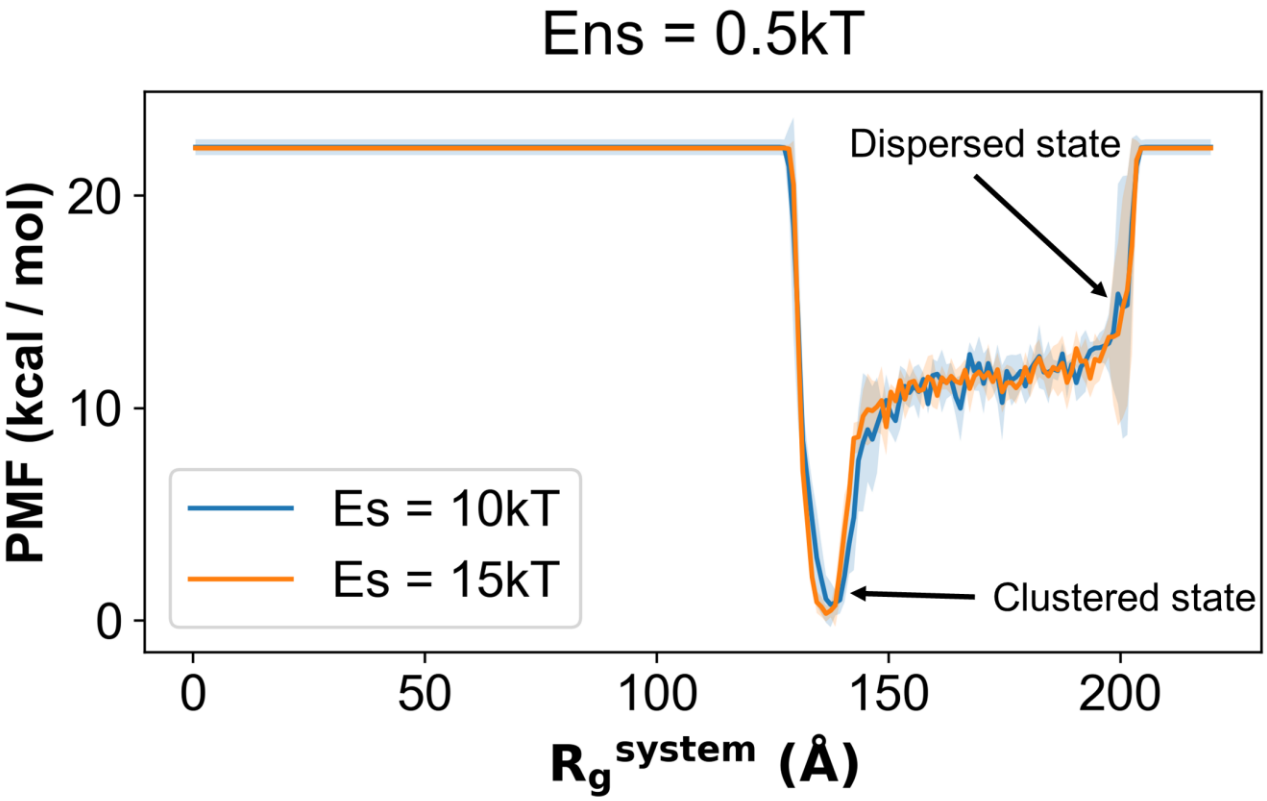

Figure S5: Free energy profile of 400 chains coalescing into one large cluster. The Rg^{system} at the minimum free energy corresponds to the fully clustered state. Each line is an average of 5 stochastic trials. The solid line is the mean and fluctuation envelop represents the standard deviation.

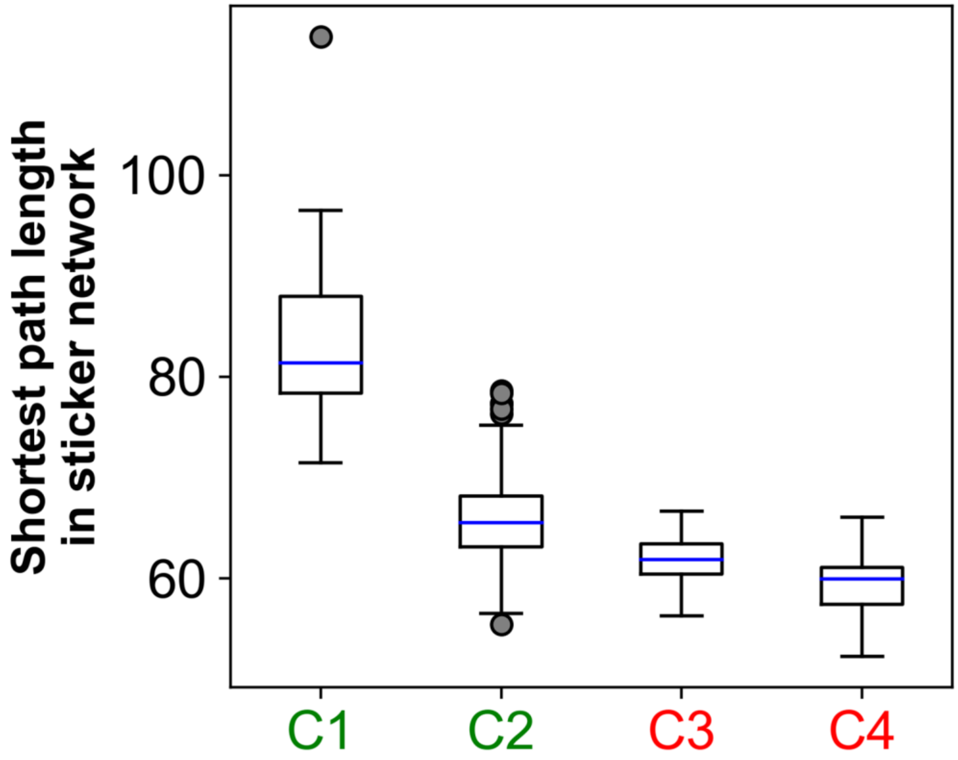

.

Figure S6: Shortest path length distribution of the sticker-spacer network. Each network (cluster) has 7000 nodes / beads (stickers + spacers). The inter-sticker bonds serve as edges. Starting from one node, the shortest topological paths to all other nodes are computed. Hence, the path length is in the unit of bead count. The color labels are same as in Figure 3. Parameter combinations (Es, Ens) are divided into two categories: C1 (10kT, 0.3kT) and C2 (10kT, 0.5kT) are labelled in green as they undergo fusion; C3 (15kT, 0.3kT) and C4 (15kT, 0.5kT) are labelled in red which do not fuse.

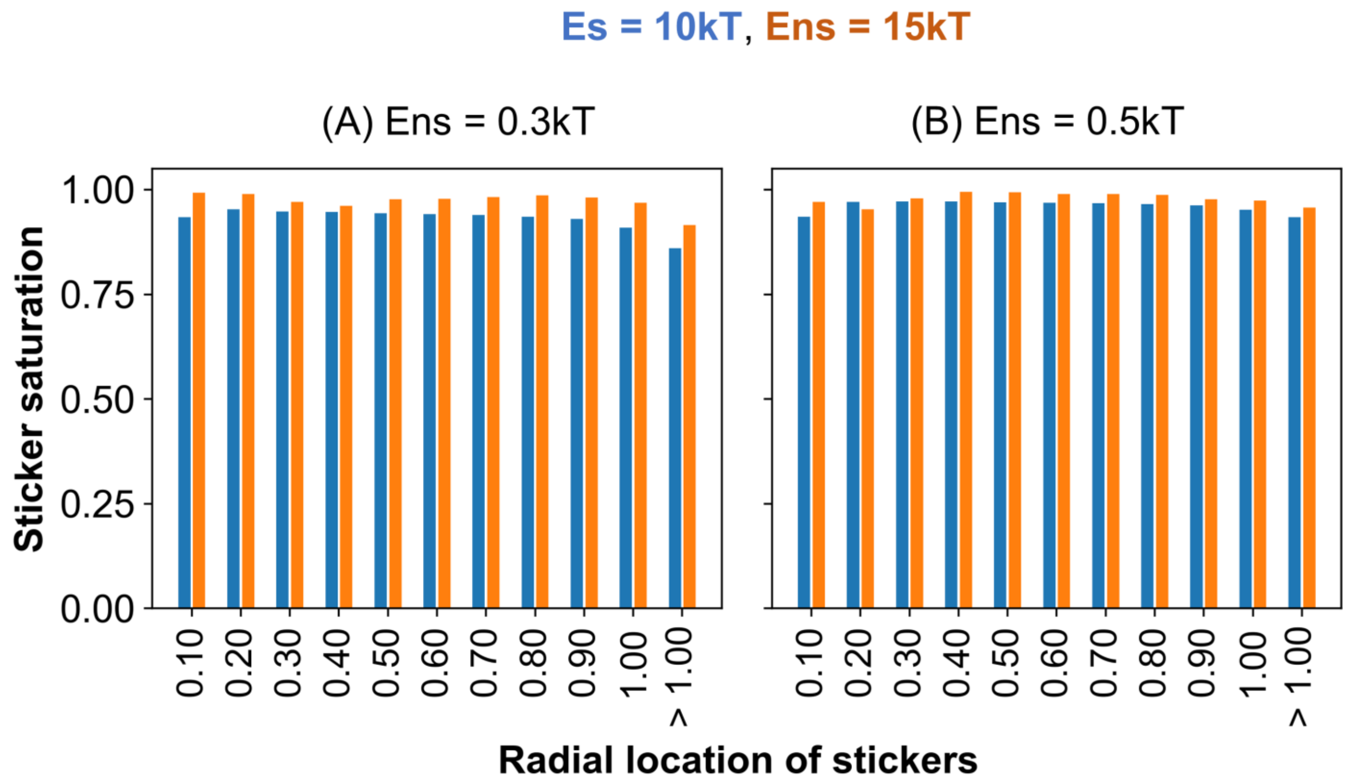

Figure S7: Spatial distribution of saturated stickers within clusters. Extent of sticker saturation as a function of their radial locations within the cluster, at (A) Ens = 0.3kT and (B) Ens = 0.5kT. The blue bars correspond to Es = 10kT, while the orange bars refer to Es = 15kT. For each condition, we compute the distance ($R_{sticker}$) of each sticker from the cluster center and normalize by cluster radius, $R_{cluster}$, where $R_{cluster} = \sqrt{\frac{5}{3}} * R_g$ and R_g is the radius of gyration of the cluster. This normalized radial location of stickers is plotted in the horizontal axis. When $\frac{R_{sticker}}{R_{cluster}} \sim 1$, stickers are located near the cluster periphery.

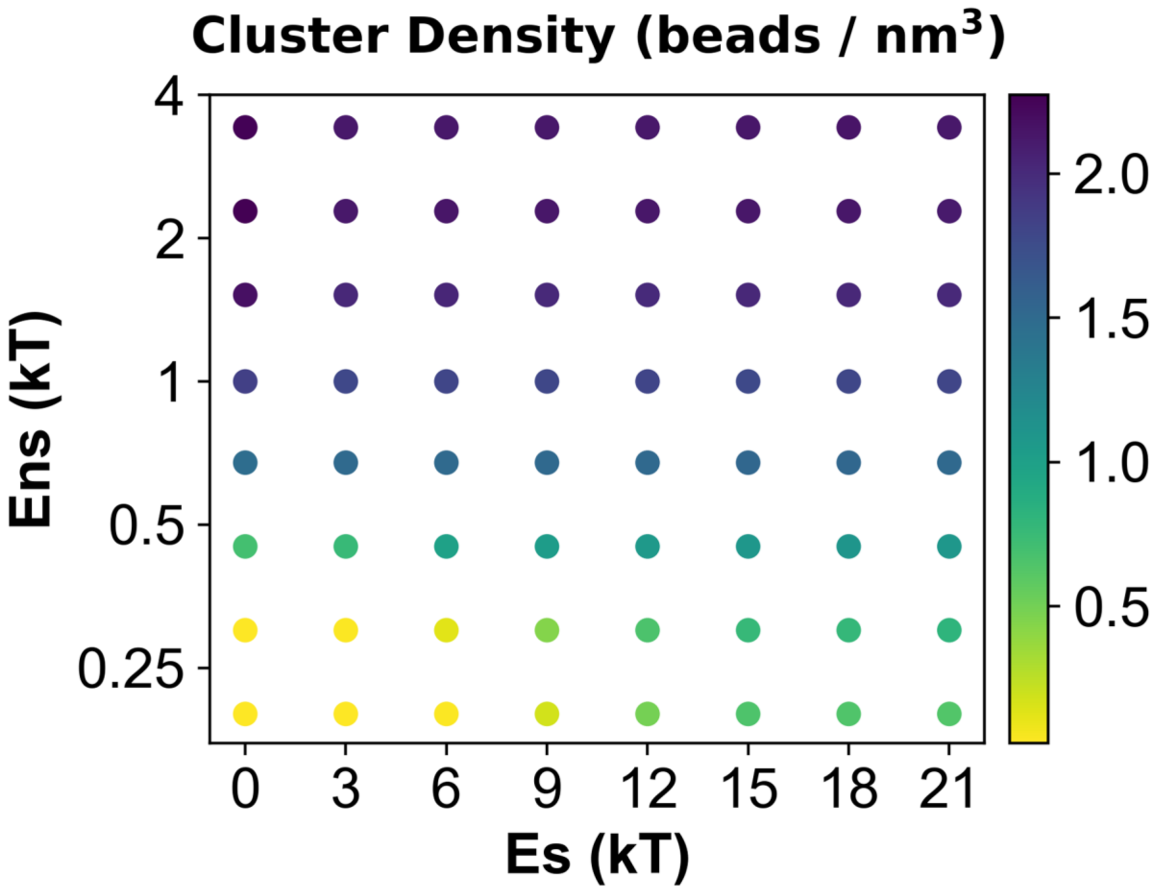

Figure S8: Quantification of cluster density across the phase diagram. Density is computed at the last timepoint of relaxation trajectory. $Density = \frac{N_{beads}}{\frac{4}{3} * \pi * R_g^3}$ where R_g is radius of gyration of the cluster and N_{beads} is the total number of beads (stickers + spacers) present in the cluster.

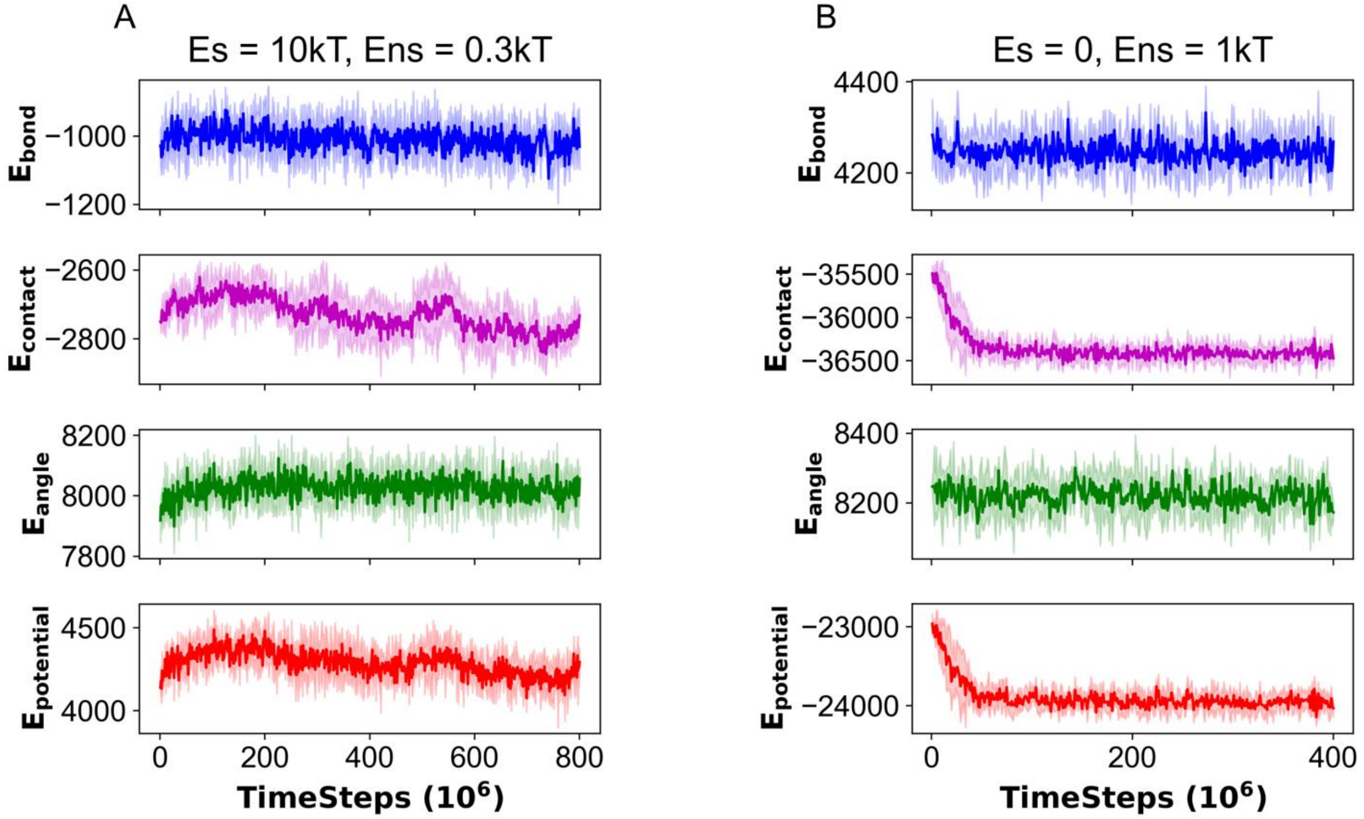

Figure S9: Comparison of energy profiles during fusion of two clusters made of (A) Heteropolymers (B) Homopolymers. E_{bond} includes all the bonds (permanent and breakable) present in the system. $E_{contact}$ refers to the sum of contact energies coming from the pairwise Lennard-Jones (non-specific) interactions. E_{angle} is angular energy. $E_{potential} = E_{bond} + E_{pair} + E_{angle}$. Energy unit is kcal/mol.

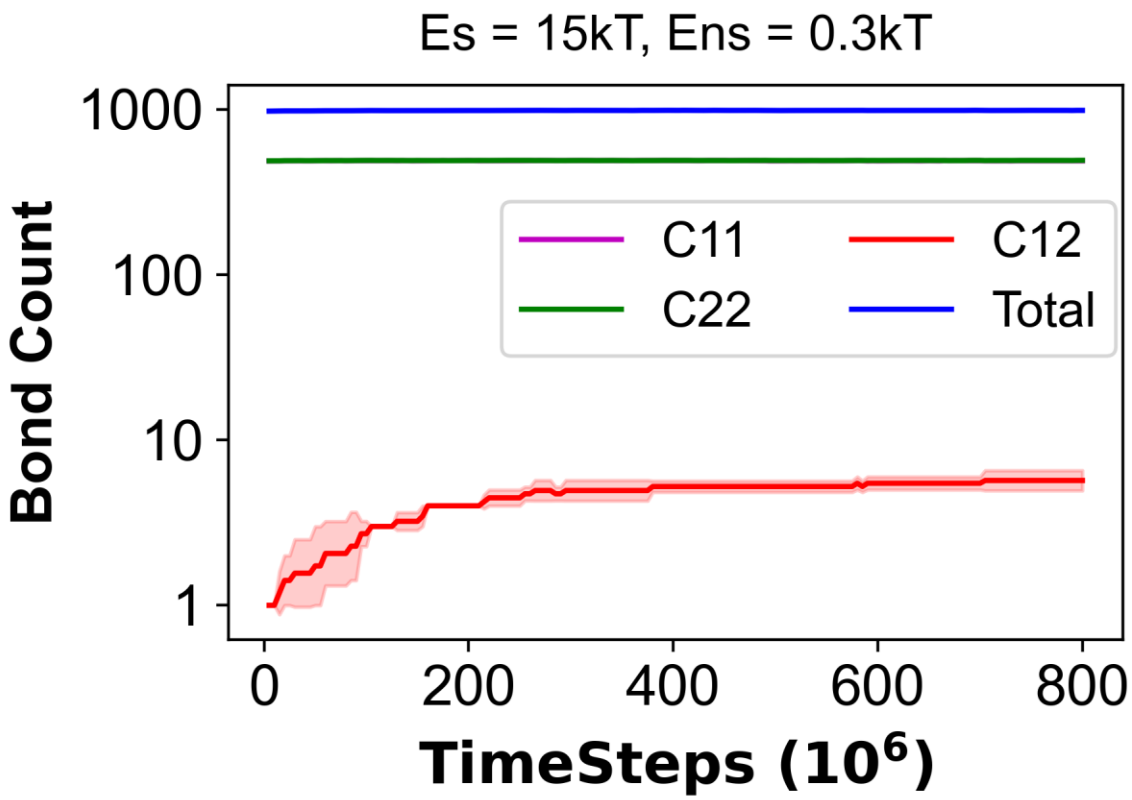

Figure S10: Lack of bond exchange triggers kinetic arrest of sticker-saturated clusters. C11, C22 and C12 indicate intra-cluster-1, intra-cluster-2 and inter-cluster, respectively. “Total” indicates the entire system.

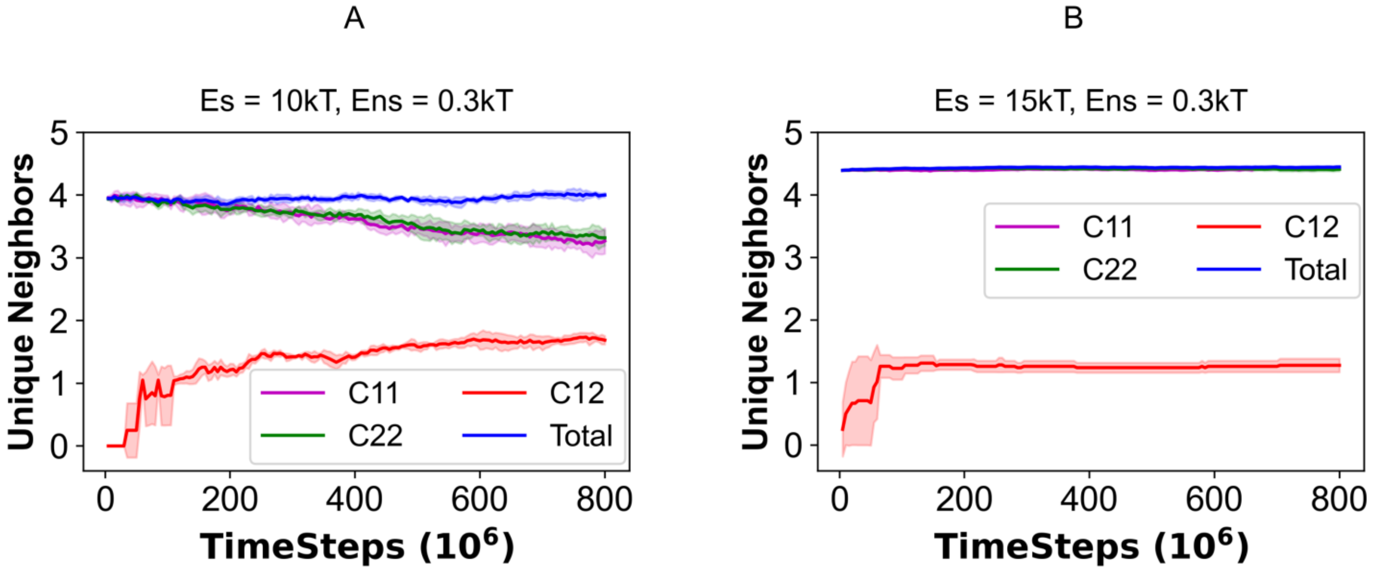

Figure S11: Neighbor exchange during cluster fusion. Since a chain contains 5 stickers, it can be bonded with one neighboring chain at minimum and five neighbors at maximum. If two chains establish two bonds between them, they still have one unique neighbor each. A free chain has no neighbor. Two energy combinations are shown when clusters (A) fuse and (B) do not fuse. C11, C22 and C12 stand for intra-cluster-1, intra-cluster-2 and inter-cluster, respectively. “Total” indicates the entire system.

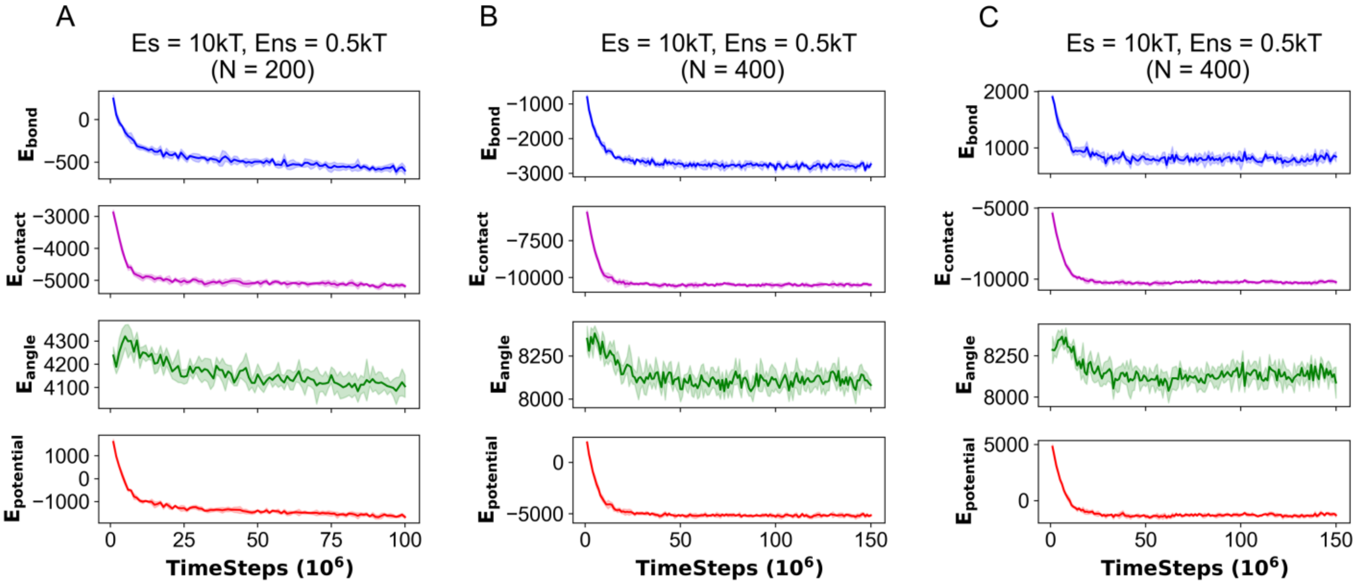

Figure S12: Energy profiles during dispersed to clustered phase transition for sticker-spacer polymers. (A) 200 chains coalescing into one large cluster at Es = 10kT, Ens = 0.5kT. (B, C) 400 chains coalescing into one large cluster at Es = 10kT and 15kT (Ens = 0.5kT) respectively. E_{bond} includes all the bonds (permanent and breakable) present in the system. $E_{contact}$ refers to the sum of contact energies coming from the pairwise Lennard-Jones (non-specific) interactions. E_{angle} is angular energy. $E_{potential} = E_{bond} + E_{pair} + E_{angle}$. Energy unit is kcal/mol. Each trajectory is an average over 5 stochastic runs (Solid line: mean, fluctuation envelop: standard deviation).

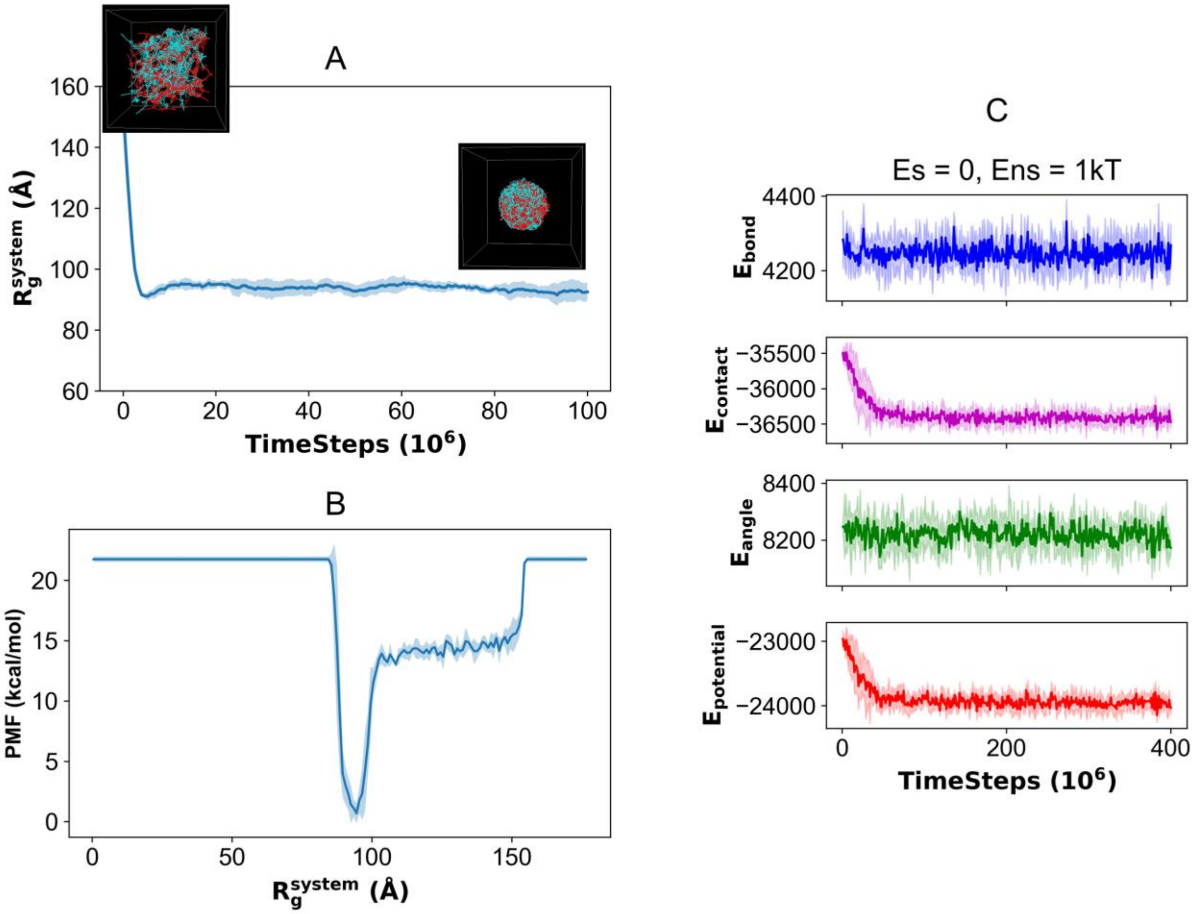

Figure S13: Clustering dynamics of homopolymers (Es = 0, Ens = 1kT). (A) Timecourse of the metadynamics order parameter R_g^{system}, as defined in Figure 1. Insets show first and last timeframes depicting dispersed and clustered states. Since Es = 0, all the beads are colored either red or cyan to indicate that all of them interact in a similar manner. We note that there is no distinction between the chain types here since Es = 0. We still use two color labels to be consistent with Figure 1 color scheme. (B) Free energy profile where the minimum corresponds to the fully clustered state. (C) Energy profile. E_{bond} includes all the bonds (permanent and breakable) present in the system. $E_{contact}$ refers to the sum of contact energies coming from the pairwise Lennard-Jones (non-specific) interactions. E_{angle} is angular energy. $E_{potential}$ = E_{bond} + E_{pair} + E_{angle}. Energy unit is kcal/mol. Each trajectory is an average over 5 stochastic runs (Solid line: mean, fluctuation envelop: standard deviation).
